# DNA damage and repair: underlying mechanisms leading to microcephaly

**DOI:** 10.3389/fcell.2023.1268565

**Published:** 2023-10-10

**Authors:** Jessica Honorato Ribeiro, Nazlican Altinisik, Nicholas Rajan, Mieke Verslegers, Sarah Baatout, Jay Gopalakrishnan, Roel Quintens

**Affiliations:** ^1^ Radiobiology Unit, Belgian Nuclear Research Centre (SCK CEN), Mol, Belgium; ^2^ Faculty of Bioscience Engineering, Ghent University, Ghent, Belgium; ^3^ Laboratory for Centrosome and Cytoskeleton Biology, Institute of Human Genetics, University Hospital, Heinrich-Heine-Universität, Düsseldorf, Germany

**Keywords:** neurodevelopment, microcephaly, DNA damage, DNA repair pathways, DNA repair deficiency, ionizing radiation, Zika virus, p53

## Abstract

DNA-damaging agents and endogenous DNA damage constantly harm genome integrity. Under genotoxic stress conditions, the DNA damage response (DDR) machinery is crucial in repairing lesions and preventing mutations in the basic structure of the DNA. Different repair pathways are implicated in the resolution of such lesions. For instance, the non-homologous DNA end joining and homologous recombination pathways are central cellular mechanisms by which eukaryotic cells maintain genome integrity. However, defects in these pathways are often associated with neurological disorders, indicating the pivotal role of DDR in normal brain development. Moreover, the brain is the most sensitive organ affected by DNA-damaging agents compared to other tissues during the prenatal period. The accumulation of lesions is believed to induce cell death, reduce proliferation and premature differentiation of neural stem and progenitor cells, and reduce brain size (microcephaly). Microcephaly is mainly caused by genetic mutations, especially genes encoding proteins involved in centrosomes and DNA repair pathways. However, it can also be induced by exposure to ionizing radiation and intrauterine infections such as the Zika virus. This review explains mammalian cortical development and the major DNA repair pathways that may lead to microcephaly when impaired. Next, we discuss the mechanisms and possible exposures leading to DNA damage and p53 hyperactivation culminating in microcephaly.

## 1 Introduction

The human genome is constantly exposed to damaging agents, resulting in around 70,000 DNA lesions per cell daily (Tubbs and Nussenzweig, 2017). Damaging agents can be either endogenous, arising from replicative stress, oxidative stress, and transcriptional activity, or exogenous sources such as ultraviolet (UV) or ionizing radiation (IR), viruses, and chemicals. The induced lesions can lead to mutations in the basic structure of the DNA, threatening genome integrity and causing a myriad of human diseases. To counteract genotoxic stress, cells have developed several mechanisms to recognize and repair the damages, collectively called the DNA damage response (DDR) (Senturk and Manfredi, 2013; Mikolaskova et al., 2018).

Among the most frequent DNA lesions are base mismatches, apurinic/apyrimidinic sites, interstrand crosslinks, bulky lesions (DNA adducts), single DNA-strand breaks (SSBs), and double DNA-strand breaks (DSBs). The presence of these lesions triggers the cellular DDR, a tightly controlled set of events defending cells facing injuries. The DDR mainly occurs through multiple and complex DNA repair pathways, activation of cell cycle checkpoints, and tolerance processes that work together to preserve genome stability (Giglia-Mari et al., 2011). The choice of the appropriate DDR will depend on different factors such as the type of lesion and the phase of the cell cycle during which it occurs (Carusillo and Mussolino, 2020).

It is known that the DDR plays a pivotal role in both the developing and mature nervous system and a defective DNA repair machinery is very often associated with neurological disorders. The classification of the diseases depends typically on their repair defect and the range of clinical features (O’Driscoll and Jeggo, 2008). In the mature brain, DDR deficiency is linked with impaired transcription, aging, and neurodegenerative diseases such as Alzheimer’s and Parkinson (McKinnon, 2009; Madabhushi et al., 2014). However, failure of DDR mechanisms in the developing brain is often associated with syndromes, including neurodevelopmental defects such as microcephaly (Aditi and McKinnon, 2022). Accordingly, among all the embryonic tissues, the developing brain is the most sensitive to DNA damage (McKinnon, 2009), especially at the early stages of neurogenesis. Therefore, it is believed that during early brain development, the neural progenitor pool responsible for expanding the neocortex can quickly deplete when facing DNA damage. Thus, the accumulation of lesions, potentially leading to cell death or reduced proliferation, becomes critical and impairs proper development (O’Driscoll and Jeggo, 2008).

Microcephaly is defined as a significantly reduced head circumference by more than two standard deviations below the mean for sex, age, and ethnicity. When the head circumference is more than three standard deviations below the mean, it is considered a severe microcephaly (DeSilva et al., 2017; Becerra-Solano et al., 2021). This disease can be classified as (Tubbs and Nussenzweig, 2017) primary microcephaly that can be diagnosed right after birth and has a non-progressive nature, or (Mikolaskova et al., 2018) secondary microcephaly that develops at later life stages and is a progressive neurodegenerative disorder (Papoulidis et al., 2022).

Microcephaly can also be categorized into (Tubbs and Nussenzweig, 2017) non-syndromic when the reduced brain volume is an isolated finding, or (Mikolaskova et al., 2018) syndromic when it is accompanied by additional features such as primordial dwarfism, radiosensitivity and chromosome breakage (Siskos et al., 2021). Currently, there are over 800 genes linked to microcephaly (Boonsawat et al., 2019). So far, 30 of these genes constitute a primary microcephaly subclass named microcephaly primary hereditary (MCPH) (Hamosh et al., 2000; Zaqout and Kaindl, 2022; OMIM, 2023), which is a non-syndromic genetic form of microcephaly that is better characterized when compared to secondary microcephaly and non-MCPH (Boonsawat et al., 2019). Although most microcephaly cases are of genetic origin, this disorder can also be induced by exposure to moderate to high doses of IR during embryonic and fetal development, drugs such as alcohol taken during pregnancy, and intrauterine infections such as the Zika virus (Jones et al., 1973; Otake and Schull, 1998; Gabriel et al., 2017). This review discusses how DNA damage accumulation during embryonic development contributes to neurodevelopmental defects, leading to microcephaly.

## 2 Neocortex development

The mammalian brain originates from the neural plate. The folding of this plate to form the neural groove followed by the neural tube marks the beginning of neurogenesis (Copp et al., 2003). The neural tube is composed of a single layer of neural epithelial cells (NECs), that are the primary progenitors from which all lineages of neural cells eventually arise (Götz and Huttner, 2005; Rakic, 2009). The NECs self-renew by symmetrical divisions, generating two identical daughter cells, ensuring the formation of a critical precursor pool size, which is one of the crucial determining factors of brain size (Götz, 2003). When the precursor pool has sufficiently expanded, the neurogenic phase starts with NECs giving rise to radial glia cells (RGCs), which are the majority of precursors populating the proliferative ventricular zone (VZ) and subventricular zone (SVZ) of the mammalian neocortex (Götz, 2003; Paridaen and Huttner, 2014). Then, a proportion of RGCs shift to asymmetric divisions, giving rise to an identical proliferating daughter cell and to an intermediate progenitor (IP) with proliferative capacity, that further expand the progenitor pool by symmetric division (indirect neurogenesis), or rarely a neuron (direct neurogenesis) (Götz, 2003; Martínez-Cerdeño and Noctor, 2018; Xing et al., 2021). Symmetric and asymmetric cell division are settled by proper spindle orientation, which is required for cell fate determination and differentiation. Changes in the orientation of the mitotic spindle can compromise proper cortical development, resulting in premature generation of neurons instead of neural progenitor cells (NPCs) expansion (Lancaster and Knoblich, 2012; Paridaen and Huttner, 2014; Mfossa et al., 2020). A detailed overview of the different types of cell division driving neurogenesis can be found in the following literature (Götz, 2003; Paridaen and Huttner, 2014; Martínez-Cerdeño and Noctor, 2018; Xing et al., 2021). During corticogenesis, most of the RGCs generate only neurons/IPs, while others are already committed to glial lineages arising during late neurogenesis, and only few NPCs are still multipotent and can give rise to both neurons/IPs and glial cells (Götz, 2003; Pilaz et al., 2016; Martínez-Cerdeño and Noctor, 2018). These immature cells will then migrate radially until they reach their final destination in the cortical plate, thus contributing to a proper cortical expansion (Paridaen and Huttner, 2014).

The cortical folding in gyrencephalic species such as humans creates a large cortical surface area in relation to their brain volume. It is believed that gyrogenesis is only possible because of the numerous cell divisions that occur during early steps of neocortex development, provided by the increased number and diversity of progenitors (Ronan and Fletcher, 2015; Garcia et al., 2018), in particular the basal and outer RGCs, that are predominant NPC types with distinct proliferative capacity that largely contribute to cortical expansion (Hansen et al., 2010; Andrews et al., 2020). Altogether, interferences on the progenitor pool amplification can lead to impairments in brain development. Microcephaly is one of the drastic outcomes that can occur due to NPC depletion, and among the potential mechanisms leading to microcephaly are: reduced proliferative capacity, prolonged mitosis, apoptosis and premature differentiation of NPCs (O’Driscoll and Jeggo, 2008; Pilaz et al., 2016; Phan and Holland, 2021).

Importantly, most of the 30 MCPH genes (Hamosh et al., 2000; OMIM, 2023) are involved in centrosome biogenesis, indicating that alterations in the number of centrosomes and spindle position control are important mechanisms leading to NPC depletion. In addition, these causative genes are also linked to the DDR, cell cycle checkpoints, microtubule dynamics, chromosomal condensation and transcriptional activity [for review, see Jayaraman et al. (2018), Je et al. (2020), and Siskos et al. (2021)]. Despite the wide range of processes from which microcephaly can arise, it has already been demonstrated that deficiencies in the DNA repair pathways leading to the accumulation of lesions in the highly sensitive developing brain can underlie most of these mechanisms (O’Driscoll and Jeggo, 2008). The DNA lesions and DDR-related pathways associated with microcephaly are described hereunder. Other mechanisms underlying primary microcephaly have been excellently reviewed elsewhere (Phan and Holland, 2021).

## 3 DNA lesions and repair pathways

As mentioned in the introduction, damage to the DNA can generate different types of lesions, of which DSBs are considered the most lethal type for cells. Indeed, a single unrepaired DSB is sufficient to trigger mutations, loss of heterozygosity, and chromosome rearrangements resulting in cell death (Wendy and Pederson, 2017). The different pathways that are implicated in DSB resolution are non-homologous DNA end joining (NHEJ), alternative non-homologous DNA end joining (alt-NHEJ), homologous recombination (HR), and single-strand annealing. NHEJ and HR are the two main cellular mechanisms to repair DSBs in eukaryotic cells (Han et al., 2017; Wendy and Pederson, 2017). For the more frequent DNA lesions, the DNA mismatch repair (MMR) pathway corrects insertion/deletion mispairs and mismatched bases; the base excision repair (BER) pathway deals with apurinic/apyrimidinic sites; the nucleotide excision repair (NER) pathway corrects bulky DNA lesions and the Fanconi Anaemia (FA) pathway deals with interstrand crosslinks. Among these pathways, NHEJ, HR, NER, and FA are known to be implicated in syndromic microcephaly and are therefore further addressed in this review (McKinnon, 2009; Brandsma and Gent, 2012; O’Driscoll, 2012; Carusillo and Mussolino, 2020).

**FIGURE 1:**
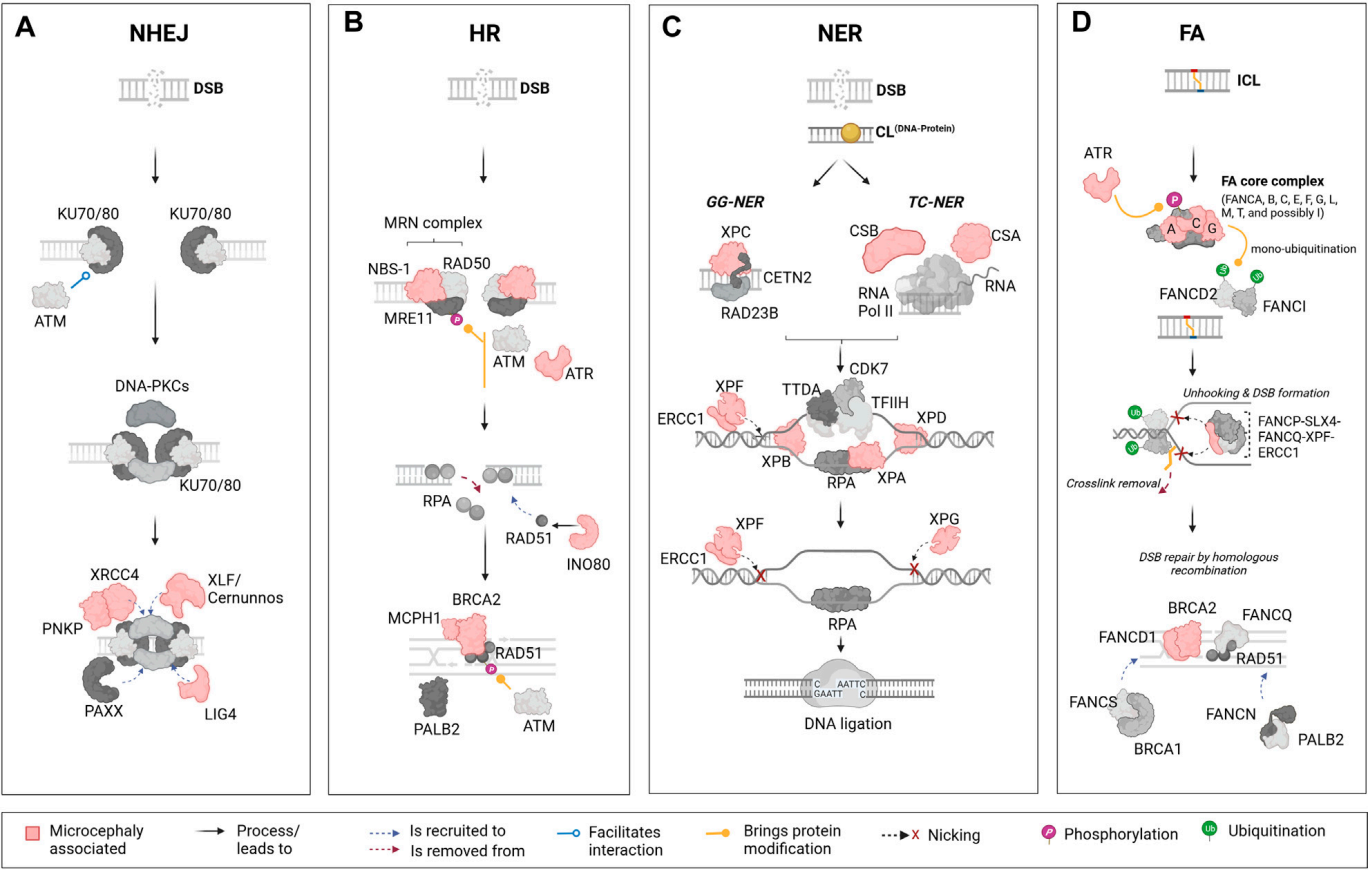
Schematic representation of the major DNA repair pathways and key components linked to microcephaly. The figure represents four key DNA damage repair pathways from left to right: the non-homologous end joining (NHEJ) pathway, homologous recombination (HR) pathway, nucleotide excision repair (NER) pathway, and Fanconi anaemia (FA) pathway. Each pathway presents its main components and their associated functional importance. Components that are linked to microcephaly upon mutation are marked in “light red” across these pathways. **(A)**: The NHEJ pathway is one of the primary mechanisms in cells to repair DNA double-strand breaks (DSBs). This pathway is distinct in that it does not require a homologous sequence to guide the repair. Mutations in certain elements of this pathways are reported to lead to microcephaly; these include X-Ray repair cross complementing 4 (XRCC4), Polynucleotide kinase phosphatase (PNKP), XRCC4-like factor/Cernunnos (XLF/Cernunnos), and DNA ligase IV (LIG4). **(B)**: In contrast to the NHEJ pathway, the HR is a DNA repair mechanism that addresses DSBs by using an undamaged, homologous DNA sequence as a template. In this pathway, mutations in components such as Nijmegen breakage syndrome 1 (NBS-1), Ataxia telangiectasia and Rad3-related protein (ATR), INO80 complex subunit D (INO80), Microcephalin 1 (MCPH1), and Breast cancer 2 (BRCA2) are reported to lead to microcephaly. **(C)**: The NER pathway is a DNA repair mechanism designed to rectify bulky, helix-distorting lesions as well as covalent DNA-protein crosslinks. In NER, damage detection proteins identify the lesion, and the surrounding DNA segment is unwound. Specialized endonucleases then excise the damaged strand, creating a gap. DNA polymerase fills in this gap using the undamaged strand as a template, and DNA ligase seals the final bond, restoring the DNA to its original state. Several components from the NER pathway are also included, such as Cockayne syndrome B (CSB), Cockayne syndrome A (CSA), Xeroderma pigmentosum groups C, F, B, A, D, G (XPC, XPF, XPB, XPA, XPD, XPG), and Excision Repair 1, Endonuclease Non-Catalytic Subunit (ERCC1). **(D)**: The FA pathway, which plays a role in the repair of interstrand crosslinks (ICLs) involves highly coordinated protein-DNA and protein-protein interactions within the nucleus, including the identification of the ICL, unhooking, and removal of the crosslink, followed by DNA repair through HR. In humans, mutations in FA groups A, C, G (FANCA, FANC, FANCG), and genes in Group D1/BRCA2 (FANCD1/BRCA2) are known to result in microcephaly in patients. Mutations in additional components of the FA pathway, such as ATR and ERCC1 are also linked to microcephaly in humans. This figure highlights the role of specific DNA repair pathways’ components in the etiology of microcephaly. The lower panel of the illustration contains descriptions for interactions and other processes or modifications that are represented by figure objects. Illustration created using Biorender (https://www.biorender.com/).

The HR pathway uses the sister chromatid as a template for repair. Therefore, it is relatively slow but mostly error-free and only functional from mid S through G2 phase. In contrast, NHEJ works by ligating damaged DNA ends without the use of a DNA template. This renders NHEJ faster, but less accurate. Overall, NHEJ is the preferred DSB repair pathway during all cell cycle stages, especially in G1 (O’Driscoll, 2012; Liu et al., 2019; Recio et al., 2019). When recruited, the NHEJ pathway (Figure 1A) works through a core complex, including DNA-PKcs, DNA ligase IV (LIG4), Ku70/80 heterodimer, X-Ray repair cross-complementing protein (XRCC4) and XLF/Cernunnos (Liu et al., 2019; Recio et al., 2019). Ku70/80 is first recruited at the DSB site protecting the DNA from end-resection. Thereafter, the C-terminal domain of Ku80 recruits DNA-PKcs forming a complex which in turn recruits XRCC4, XLF/Cernunnos and LIG4 to seal the gap. At this step, a XRCC4-LIG4 complex also interacts with several DNA end-processing proteins such as the polynucleotide kinase phosphatase (PNKP) (Aceytuno et al., 2017; Carusillo and Mussolino, 2020).

The HR pathway (Figure 1B) processes DSBs into single-stranded DNA (ssDNA) in an initial step involving proteins of the MRN complex (MRE11-RAD50-NBS1). ssDNA are promptly bound by the replication protein A (RPA) complex (RPA1, RPA2 and RPA3). Next, the DNA Repair Protein 52 removes RPA allowing its replacement by DNA Repair Protein 51 (RAD51) via a Breast Cancer Associated Gene 2 (BRCA2)-dependent process. The RAD51-ssDNA complex formation is crucial for the next steps of HR repair, mediating the search for homologous sequences on the sister chromatid to generate the new DNA sequence (Krajewska et al., 2015; Ma et al., 2017; Carusillo and Mussolino, 2020).

There are mainly two mechanisms of DNA damage repair by NER (Figure 1C). In the global-genome nucleotide excision repair (GG-NER) pathway, the lesions are removed throughout the genome whereas the transcription-coupled nucleotide excision repair (TC-NER) refers to the faster removal of damage from the transcribed strands of active genes (Marteijn et al., 2014; Carusillo and Mussolino, 2020). Mechanistically, GG-NER and TC-NER differ only in the first step, which is the detection of lesions. The GG-NER pathway employs XPC, in association with hRAD23B and centrin 2, to directly identify a lesion. On the other hand, TC-NER is initiated after RNA polymerase II (RNAPII) is blocked by discontinuities in the template strand caused by bulky adducts, followed by the recruitment of TC-NER-specific factors like CSB and the CSA complex. Following successful damage recognition, the next steps are identical for both pathways, involving the recruitment of the transcription initiation factor IIH (TFIIH) complex. Along with the scaffolding protein XPA and replication protein A, TFIIH unwinds the DNA, creating a precision DNA bubble that is recognized by repair endonucleases ERCC1-XPF and XPG that cleave the damaged strand and gap-filling DNA synthesis is then facilitated by DNA polymerases, which use undamaged NTS as a template (Scharer, 2013; Petruseva et al., 2014; Duan et al., 2021).

Lastly, the studies about an autosomal recessive disease named Fanconi Anaemia brought to light the existence of a pathway to repair interstrand crosslinks. The FA repair (Figure 1D) is a highly complex pathway involving FA and non-FA proteins, and other repair pathways such as HR, NER and translesion synthesis. To date, 23 FA genes have been identified, coding for proteins that can be divided in three different groups: (Tubbs and Nussenzweig, 2017) the FA core complex composed by 8 FANC proteins (A-C, E-G, L-M) and FAAP associated proteins; (Mikolaskova et al., 2018) the FANCD2/FANCI complex; and (Senturk and Manfredi, 2013) the FANC effector proteins (D1, J, N-S). The core complex is responsible for FANCD2/FANCI ubiquitination, a critical step that allows FANCD2/FANCI recruitment to the lesion site. Once there, this complex orchestrates the recruitment of effector proteins working on unlinking crosslinked bases, inserting new bases, precise DNA end resection and lesion repair and removal (Lopez-Martinez et al., 2016; Carusillo and Mussolino, 2020; Repczynska et al., 2022).

As exemplified above, the DDR pathways rely on a complex crosstalk between a wide range of proteins participating in different steps to achieve DNA repair and contribute to genomic stability. Hereunder we will discuss how genetic mutations and environmental factors can disturb these pathways to impair proper brain development and drive microcephaly.

## 4 Microcephaly associated with DNA damage

Genetic mutations are the leading cause of microcephaly in humans and are often associated with alterations in genes that play a role in the repair of DSBs and SSBs. Syndromic patients usually show genomic instability and sensitivity to IR (Li et al., 2008a; Aditi and McKinnon, 2022). The impaired brain development commonly observed in damage response syndromes suggests that the capability to respond to endogenous DNA damage is essential for maintaining proper development (Mao et al., 2008; Liu et al., 2019). It is known that mutations in different components/regulators of the two main pathways responsible for repairing DSBs, NHEJ and HR, can lead to a range of human syndromes/diseases such as LIG4, XLF/Cernunnos, XRCC4 (Menon and Povirk, 2017; Roch et al., 2021), Nijmegen breakage (NBS), Ataxia-telangiectasia and Rad3-related protein (ATR) Seckel (ATR-SS), primary microcephaly 1 (MCPH1), respectively (Wu et al., 2009; García-de Teresa et al., 2017). For the less frequent lesions, mutations in the NER pathway can induce Xeroderma Pigmentosum (XP), Cockayne syndrome (CS) and Trichothiodystrophy (TTD). Mutations in the FA pathway can lead to Fanconi anemia (Kraemer et al., 2007; Lopez-Martinez et al., 2016). All of these and other relevant microcephaly-related diseases/syndromes will be discussed hereunder in more detail and a summary can be found in Tables 1–3.

**TABLE 1:** Symptomatology and microcephaly mechanistic of NHEJ deficiency.

Human patients						
NHEJ factor	Microcephaly	Radiosensitivity	Developmental delay	Immunodeficiency	Seizures	Predisposition to cancer
XLF	Yes	Yes	Unknown	Yes	No	No
LIG4	Yes	Yes	Yes	Yes	No	Yes
XRCC4	Yes	Yes	Yes	No	No	No
PNKP	Yes	Yes	Yes	No	Yes	No

Mouse models						
NHEJ factor	Viability	Radiosensitivity	Affected brain cells	Mechanism		
Xlf	Viable	Yes	NPCs, newborn neurons	Premature differentiation, mild apoptosis		
Lig4	Lethal	Yes	Newborn neurons	p53-dependent apoptosis		
Xrcc4	Lethal	Yes	Newborn neurons	p53-dependent apoptosis		
Pnkp	Lethal	Yes	NPCs, mature neurons, oligodendrocytes	p53-dependent apoptosis		

**TABLE 2:** Symptomatology and microcephaly mechanistic of HR deficiency.

Human patients					
NHEJ factor	Microcephaly	Radiosensitivity	Developmental delay	Immunodeficiency	Predisposition to cancer
NBS-1	Yes	Yes	Yes	Yes	Yes
ATR	Yes	Yes	Yes	No	Yes
BRCA2	Yes	Yes	Yes	No	Yes
MCPH1	Yes	-	Yes	No	Yes
INO80	Yes	-		No	No

Mouse models					
NHEJ factor	Viability	Radiosensitivity	Affected brain cells	Mechanism	
Nbs-1	Lethal	Yes	NPCs, newborn neurons	Premature differentiation and p53-dependent apoptosis	
Atr	Viable	Yes	NPCs	p53-dependent apoptosis	
Brca2	Lethal	Yes	NPCs, newborn neurons	p53-dependent apoptosis	
Mcph1	Viable	Yes	NPCs	premature differentiation	
Ino80	Lethal	-	NPCs	p53-dependent apoptosis	

**TABLE 3:** Symptomatology and microcephaly mechanistic of NER deficiency.

Human patients					
NER factor	Microcephaly	Radiosensitivity	Developmental delay	Immunodeficiency	Predisposition to cancer
XPA	Yes	Yes	Yes	Yes	Yes
XPB	Yes	Yes	No	-	Yes
XPC	Yes	Yes	No	Yes	Yes
XPD	Yes	Yes	Yes	Yes	Yes
XPF	Yes	Yes	Yes	-	-
XPG	Yes	Yes	Yes	-	-
CSA	Yes	Yes	Yes	No	No
CSB	Yes	Yes	Yes	No	No

Mouse models					
NER factor	Viability	Radiosensitivity	Affected brain cells	Mechanism	
Csa	Viable	Yes	-	-	
Csb	Viable	Yes	-	-	
Csb/Xpa	Lethal	Yes	Cerebellar external granular layer, Purkinje cells	Decreased neuronal proliferation, increased apoptosis	
Csb/Xpc	Lethal	Yes	Cerebellar external granular layer, Purkinje cells	Decreased neuronal proliferation, increased apoptosis	

### 4.1 NHEJ pathway related syndromes/diseases

LIG4 and XLF/Cernunnos syndromes are rare autosomal recessive disorders with symptoms including microcephaly, severe growth delay, “bird-like” facial appearance, bony malformations, immunodeficiency and increased cellular sensitivity to IR (Çipe et al., 2014; Altmann and Gennery, 2016; Recio et al., 2019; Gerasimou et al., 2020). These shared features are due to loss of LIG4 and NHEJ1 function in encoding key NHEJ repair elements, impairing the final rejoining. The third component that makes up the final step of rejoining is XRCC4. Phenotypically, mutations in *XRCC4* can induce microcephaly and growth delay (Guo et al., 2015; Rosin et al., 2015; Altmann and Gennery, 2016). Mutations in *LIG4* are characterized by non-progressive microcephaly accompanied by immunodeficiency with or without neurodevelopmental delay (Altmann and Gennery, 2016). The evident prenatal microcephaly phenotype may arise due to ROS production in the fetal brain, inducing an accumulation of unrepaired DSBs (Altmann and Gennery, 2016; Lun et al., 2019).

Another hypothesis is that DSB formation may facilitate the expression of early response genes, which are markers of neural activity and insults (Hou and MacManus, 2002; Javed et al., 2018). In both cases, the neuronal population would be predisposed to apoptosis halting brain development without proper NHEJ function (Altmann and Gennery, 2016; Lun et al., 2019). Accordingly, *Lig4* deficiency in mice is described as a cause of drastic cell death of newborn neurons and embryonic lethality, which may result from a sensitivity to unrepaired DSBs as observed in the human disease (Gao et al., 1998; Frank et al., 2000). Neuronal death was observed in the entire cortex; however, the lower cortical layers were more severely affected, indicating that *Lig4* plays a role in the maintenance of a particular subset of neurons populating the radial extent of the cerebral cortex (Lun et al., 2019). Consistent with the role of LIG4 in DSB repair, different *Lig4* models showed a marked sensitivity to IR as observed in syndromic patients (Adachi et al., 2001; Hentges et al., 2006; Gatz et al., 2011). In a LIG4 (*Lig4* ^Y288C^) mouse model analyzed for its susceptibility to IR, microcephaly was proposed to occur due to apoptosis resulting from persisting DSBs in the intermediate zone arising from the transit of damaged cells coming from the VZ and SVZ. This study also proposed that IR-induced apoptosis in the VZ and SVZ is partly ataxia telangiectasia mutated (ATM)-dependent, while in the intermediate zone, it is entirely ATM-dependent (Gatz et al., 2011). Signals generated by DNA damage occurrence, especially DSBs, are known to activate p53 through the ATM signaling (Canman et al., 1998; Frank et al., 2000).

Interestingly, in DDR-associated syndromes where patients display a mutation in the *ATM* component, microcephaly is not commonly observed, most likely because p53 cannot be activated (O’Driscoll and Jeggo, 2008). The activation of a p53-dependent apoptosis in response to DSB accumulation was suggested to underlie the *Lig4* deficiency, as indicated by a rescue of neuronal death and embryonic lethality in *Trp53* ^−/−^; *Lig4* ^−/−^ mice (Frank et al., 2000). Signs of premature neuronal differentiation or disrupted neuronal migration were not apparent, suggesting that the microcephalic phenotype was completely apoptosis-dependent (Gao et al., 1998; Lun et al., 2019).

Deleterious mutations in *XLF/Cernunnos* were identified for the first time in 2006, where all patients presented developmental defects including microcephaly (Ahnesorg et al., 2006; Buck et al., 2006). Curiously, in contrast to *Lig4* KO mice, *Xlf/Cernunnos* KO mice are viable without gross developmental defects, limited to a few apoptotic cells in the embryonic brain (Vera et al., 2013; Abramowski et al., 2018), suggesting only minor DDR defects (Mokrani et al., 2020). However, radiosensitivity is still common in different *Xlf*-deficient models, such as mouse embryonic fibroblasts, pro-B cell lines and embryonic stem cells (Zha et al., 2007; Li et al., 2008b). Comparable to LIG4 syndromic embryos, mice deficient for both *Xlf* and *Paxx*, a recently identified component of the NHEJ process, presented embryonic lethality and extensive p53-dependent cell death, which was not the case for single *Paxx* or *Xlf* KO mice (Abramowski et al., 2018; Castañeda-Zegarra et al., 2019). It is believed that the difference between the human and mouse XLF phenotypes may be because different NHEJ and DDR factors such as ATM, H2A.X, 53BP1 and DNA-PKcs could partially compensate for the *Xlf* deficiency in mice, avoiding major developmental impairments (Gago-Fuentes and Oksenych, 2021). Yet, strikingly, another study by Bery et al. (2023), did show significant neurodevelopmental impairments in *Xlf* KO pups, with a concurrent mild microcephalic phenotype and intellectual disability, suggested to be caused by a lowered neuronal production and apoptosis of newly born neurons. Besides apoptosis, reduced proliferation and premature differentiation of *Xlf* KO RGCs could also be observed during the early stages of neurogenesis. The latter being a well-established mechanism contributing to microcephaly, was caused by an early switch from symmetric proliferative divisions to asymmetric neurogenic divisions, and considered as the primary cause for neuronal loss in *Xlf* KO mice (Mfossa et al., 2020; Bery et al., 2023). Recently, *Xlf* was pointed as an important factor for an efficient early stage development of neural progenitors in mice, as cells lacking *Xlf* presented reduced proliferative and self-renew capacity, that was intensified in cells lacking both *Xlf* and *Paxx* (Gago-Fuentes and Oksenych, 2021).

XRCC4 is an essential factor in NHEJ repair, which can directly bind to the DNA and form a stable complex with LIG4 stimulating its joining activity (Ruis et al., 2020). Homozygous missense mutations in *XRCC4* can induce severe microcephaly (Shaheen et al., 2014; Bee et al., 2015; de Bruin et al., 2015; Guo et al., 2015; Murray et al., 2015; Rosin et al., 2015). However, *XRCC4* deficiency does not entirely mimic LIG4 syndrome but also shares similarities with Cockayne syndrome, such as progressive neuronal degeneration, ataxia and no clinical immunodeficiency (Guo et al., 2015). In general, patients harboring *XRCC4* mutations mainly show microcephaly, short stature, mental disabilities and facial dysmorphism (Saito et al., 2016). *In vitro* models have shown that fibroblasts established from *XRCC4*-deficient patients presented high sensitivity to IR, defective DSB repair and increased apoptosis after DNA damage (Guo et al., 2015; Murray et al., 2015; Rosin et al., 2015). In mice, *Xrcc4* deficiency (*Xrcc4* ^−/−^) induces late embryonic lethality at the embryonic day (E) 16.5 which was attributed to massive p53-dependent apoptosis of newborn neurons resulting from increased numbers of unrepaired DSBs (Gao et al., 1998; Gao et al., 2000). Apoptosis of newborn neurons is a feature shared between *Xrcc4* and *Lig4* KO mice, but not the other NHEJ-deficient mouse models, and this drastic phenotype observed in *Xrcc4* deficient mice may partly result from the associated loss of Lig4, as Xrcc4 is compulsory for its stabilization (de Villartay, 2015). In accordance, a recent study of a knock in *Xrcc4* ^*M61R*^ mouse model avoiding *Lig4* disruption showed rescued embryonic lethality and only a modest increase in apoptotic cells in the intermediate zone, indicating that microcephaly in XRCC4 syndrome may arise due to LIG4 destabilization (Roch et al., 2021).

Furthermore, it is known that NHEJ-related microcephalic syndromes can also be induced by mutations in the repair factor *PNKP*, which is mainly associated to two different syndromes: the microcephaly with seizures, hyperactivity, and developmental delay (MCSZ) and microcephaly associated with neurodegeneration and polyneuropathy (Shen et al., 2010; Poulton et al., 2013; Taniguchi-Ikeda et al., 2018). Among the NHEJ-related symptoms, seizures are an exclusive feature of MCSZ (Shen et al., 2010). PNKP is a bifunctional enzyme participating as a key factor in both BER by interacting with XRCC1 and NHEJ by interacting with XRCC4, participating in the resolution of SSBs and DSBs, respectively. Mutations in *PNKP* have been associated with DSBs accumulation (Weinf et al., 2011; Aceytuno et al., 2017; Dumitrache and McKinnon, 2017). Given the importance of PNKP in both pathways, it would be expected that its deficiency would impact beyond the brain as occurs in other repair-related syndromes such as NBS1 and LIG4, suggesting a role for PNKP in resolving specific types of DNA lesions in the human brain (Girard et al., 2004; Ben-Omran et al., 2005; Shimada et al., 2015). Strikingly, a recent study highlighted the essential role of Pnkp during mouse embryogenesis and neurogenesis, showing that both *Pnkp* deletion embryo-wide (*Pnkp* ^*Sox2-cre*^) or specifically in the brain (*Pnkp* ^*Nes-cre*^) induced lethality. Moreover, loss of *Pnkp* proved to be more severe when compared to *Lig4* or *Xrcc1* loss, indicating that at least in mice, *Pnkp* may be involved in the repair of a wider range of lesions, demonstrating an important difference between PNKP function in human and mouse (Shimada et al., 2015). In general, *Pnkp* loss has been shown to induce a thinning in the cerebral cortex, abundant p53-dependent apoptosis and impaired NPCs proliferation, leading to microcephaly (Shimada et al., 2015; Shin et al., 2021).

### 4.2 HR pathway related syndromes/diseases

Mutations in the HR pathway are responsible for a range of microcephaly-related syndromes. The main HR effectors are the MRN proteins (MRE11-RAD50-NBS1), and mutations in this complex can lead to an interaction impairment failing to process DSB (Uziel et al., 2003). All patients carrying a mutation in *NBS-1* present reduced brain size, that can have a progressive nature during the first months of life, showing that not all patients are microcephalic at birth (Hasbaoui et al., 2020). Similar to LIG4 and XLF/Cernunnos syndromes, besides marked microcephaly, “bird-like” facial features, developmental delay, immunodeficiency, radiosensitivity, and predisposition to cancer are present (Altmann and Gennery, 2016; Komatsu, 2016). At a cellular level, failure in HR repair due to NBS-1 dysregulation has been linked to genomic instability with impaired cell cycle kinetics and induction of apoptosis (Wan et al., 2013). Inactivation of *Nbs-1* in the mouse brain (*Nbn* ^*+/Δ6*^) led to decreased NPC proliferation and enhanced apoptosis of postmitotic neurons in the cerebellum through p53 activation (Frappart et al., 2005), while others showed that deletion of *Nbs-1* in the mouse brain (*Nbs1* ^*fl/fl*^; *Atr* ^*+/fl*^; NesCre+) mainly disrupts the VZ affecting only NPCs, resulting as well in decreased proliferation and apoptosis (Zhou et al., 2012). NPCs were also disturbed in *NBS-1* patient-derived brain organoids, where premature neuronal differentiation was found, accompanied by impaired DDR, high levels of yH2A.X and consequent genomic instability. The premature differentiation of NPCs and increased neuronal apoptosis are suggested to be mainly governed by the delayed ATM-p53-mediated response often observed in NBS upon endogenous DNA damage, highlighting the role of p53 in the cell fate of NPCs (Martins et al., 2022).

Seckel syndrome (SS) is a rare disorder occurring due to mutations in more than 10 genes, classifying SS as a genetically heterogeneous disease. These genes are mainly associated with the DDR or centriole formation, and syndromic patients are characterized by drastic microcephaly with mental disability, dwarfism, and growth retardation (Panigrahi et al., 2009; Kirtay et al., 2021). In 2000, *ATR* was the first gene to be mapped to chromosome 3q22.1-q24 (SCKL1) in two Pakistani families (Panigrahi et al., 2009). As mentioned before, ssDNA is one of the products resulting from DSB processing during HR, leading to the ATR activation (Krajewska et al., 2015). Besides maintaining replication fork stability, ATR coordinates cell cycle checkpoint activation, supporting genome integrity (Llorens-Agost et al., 2018). *In vitro* studies demonstrated an impaired DDR in cell lines established from severely affected individuals, mainly showing reduced phosphorylation of H2A.X, p53, and Chk1, failure to start the G2/M checkpoint, and centrosome deficiencies (O’Driscoll et al., 2003; Alderton et al., 2004). In mice, the replicative stress was further investigated, demonstrating a dramatic accumulation of γH2A.X and activated p53 throughout the embryo, culminating in increased apoptosis, aggravated by loss of p53, and eventually microcephaly. The observed high sensitivity to replicative stress in the embryonic brain could be explained by the exponential replicative expansion in the first days of life (O’Driscoll and Jeggo, 2008; McKinnon, 2009; Murga et al., 2009).

Besides the role of BRCA2 as a tumor suppressor and cell cycle regulator, it is also a key component of the HR repair pathway (Prakash et al., 2015). BRCA2 directly interacts with RAD51 through its carboxyl terminus, allowing its translocation to DSB processing sites facilitating the DNA damage repair (Holloman, 2011). Mutations in *BRCA2* leading to a complete loss of function cause embryonic lethality in both humans and mice. On the other hand, biallelic hypomorphic mutations in *BRCA2* have been associated to hypersensitivity to DNA damage and FA leading to the manifestation of microcephaly in affected patients (Weinberg-Shukron et al., 2019; Kennedy and D’Andrea, 2005; Frappart et al., 2007; Sharan et al., 1997). In mice, inactivation of *Brca2* (*Brca2* ^LoxP/LoxP^; Nestin-cre) led to increased levels of yH2AX foci followed by apoptosis in both NPCs and early post-mitotic neurons, that culminates in defective neural development leading to microcephaly due to genotoxic stress. p53 inactivation could rescue the reduced brain size, however some cell death could still be observed in the cerebellum of *Brca2* ^Nes-cre^; *Trp53* ^−/−^ mice indicating that apoptosis is partially independent of p53 (Frappart et al., 2007).

The first *Microcephalin 1* (*MCPH1*) mutation was reported in 2002 in two Pakistani families (Jackson et al., 2002). Until now, 14 additional mutations have been described, all of them leading to MCPH1, an uncommon heterogeneous disorder affecting brain development, and implicated in ATM/ATR-dependent DDR, HR, and G2/M checkpoint arrest. All these mutations are located in exons 1 to 6, and mutations found in exons 2 and 3, which encode the N-terminal BRCT domain, indicate an essential function of this domain in the neurodevelopment (Liu et al., 2016). Further reports have shown different outcomes depending on the type of mutation. A mild cellular and clinical phenotype is observed in the case of a missense mutation.

In contrast, mental disabilities and mild microcephaly are seen in the case of larger mutations affecting the six exons of *MCPH1* (Trimborn et al., 2005; Garshasbi et al., 2006; Mahmood et al., 2011). Individuals presenting mutations in the *MCPH1* gene have a head circumference below the mean, mental disabilities, developmental delays, impaired language skills and infertility, and premature chromosome condensation is considered a hallmark of this disease (Alderton et al., 2006; Wood et al., 2007; Gruber et al., 2011; Liu et al., 2021; Papoulidis et al., 2022). Mutations in *MCPH1* are proposed to link impaired DDR and MCPH occurrence (Mahmood et al., 2011). Over the past years, different genetic mouse models have been developed aiming to understand the role of MCPH1. The first *Mcph1* KO model (exon 2 deletions) survived until adulthood but presented sterility and impaired growth, with only 80% of the body weight compared to WT mice (Liang et al., 2010). Mice with an insertion between exons 3 and 4 exhibited a 15% reduction in brain weight without showing general growth retardation (Chen et al., 2013). In yet another model, a deletion of exons 4 and 5 resulted in a reduced brain weight, increased apoptosis in the VZ and SVZ, and thinning of the neocortex. A similar cortex thinning and overall reduced brain size were also observed in *Mcph1* mutant mice with a deletion of the N-terminal BRCT domain, accompanied by NPC depletion and premature differentiation (Trimborn et al., 2010). However, these manifestations were not observed after deletion of the C-terminal BRCT domain, suggesting that the DDR remained unaffected in that case. Thus, N-terminal BRCT appears to be required for a proper DDR initiation, as well as for ensuring an appropriate size of brain and regulating NPC fate (Trimborn et al., 2010; Liu et al., 2021). MCPH1 has been recognized as an essential player of the HR pathway through direct interaction with BRCA2 and RAD51, enabling the RAD51/BRCA2 complex to bind to damaged strands (Chang et al., 2020). Moreover, Zhou et al. (2013) also investigated the sensitivity of *Mcph1* KO mice to IR, revealing massive apoptosis specifically in NPCs and their immediate progeny, reinforcing the notion that a proficient DDR is crucial during early neurogenesis. Altogether, these studies show that *MCPH1* is essential for various biological processes, including DNA damage repair, chromosome condensation, and brain development.

The *INO80* gene was identified as another candidate for microcephaly and it has been shown to play a role in the HR pathway, mediating the removal of histone subunit H2A.Z, and exchanging RPA for RAD51, which are crucial steps in the DSB repair (Shaheen et al., 2014; Alatwi and Downs, 2015; Alazami et al., 2015; Morrison, 2017; Keil et al., 2020). *Ino80* conditional KO mice further revealed DSB accumulation, particularly in NPCs, triggering p53 target gene activation and apoptosis, culminating in microcephaly. Different outcomes to the loss of *Ino80* could be observed. *Ino80* deletion during NPCs symmetric divisions led to DNA breaks, which was not observed for asymmetric divisions. These findings are consistent with the possibility that activation of HR is not preferable during asymmetric divisions, being selectively activated during symmetric divisions (Keil et al., 2020).

### 4.3 NER pathway related syndromes/diseases

NER deficiencies are implicated in various autosomal recessive human disorders, including XP, CS, and TTD, which exhibit photosensitivity, skin cancer, developmental and neurological abnormalities (Vermeulen and Fousteri, 2013). Although impaired NER can explain skin sensitivity, the direct connection between UV-induced defects in neurological disorders remains unclear.

Xeroderma Pigmentosum is a rare autosomal disorder that arises from mutations in one of seven XP genes (*XPA through* -*XPG*), and the XP variant gene (Brooks, 2002). Approximately 25% of individuals with Xeroderma Pigmentosum have neurologic manifestations, including acquired microcephaly and progressive cognitive impairment. XP-A, XP-B, XP-D, XP-G, and XP-F patients have defects in NER and translesion DNA synthesis processes, making them more susceptible to neurological symptoms (Fassihi et al., 2016). In animal models, research on *Xpa*‐deficient murine neuroblasts has revealed disruptions in cellular mobility, providing a plausible mechanistic link to perturbed neurogenesis and the consequent manifestation of microcephaly (Takeuchi et al., 2023).

Cockayne syndrome is a rare autosomal recessive disorder with elevated UV sensitivity, severe growth impairment, premature aging, and progressive nervous system abnormalities (Jaarsma et al., 2013). The neurological symptoms include substantial growth defects, neuronal loss, calcification, mental retardation, and postnatal microcephaly. The severe cerebral white matter atrophy observed in CS patients is the cause of microcephaly, which is considered a cardinal feature of this syndrome (Baraitser et al., 1983; Nance and Berry, 1992; Spivak, 2004). Genetically, CS arises from mutations in five specific genes: *CSA*, *CSB*, *XPB*, *XPD*, and *XPG* (Jaarsma et al., 2013).

While murine *Csa* and *Csb* deficiencies result in only mild neurodegenerative changes, recent studies in human NPCs demonstrate that *CSB* suppression impairs neuronal differentiation, reduces microtubule-associated protein 2 (MAP2), disrupts cell polarization, and induces neuronal loss via DNA damage accumulation (Scheibye-Knudsen et al., 2012; Ciaffardini et al., 2014). These findings underscore CSB’s specific role in human postnatal neurogenesis, potentially explaining certain neurological features, such as microcephaly, in Cockayne syndrome patients (Ciaffardini et al., 2014).

Trichothiodystrophy is a clinically and genetically heterogeneous group of rare autosomal recessive disorders characterized by brittle hair, and scaly skin, including neurological defects (Price et al., 1980). It is associated with genetic mutations in the subunits of TFIIH, such as *ERCC2/XPD*, *ERCC3/XPB*, and *GTF2H5/TTDA/p8*, as well as in *MPLKIP/C7ORF11*, *RNF113A*, *GTF2E2*, *CARS1*, *TARS1*, *AARS1*, and *MARS1* genes (Theil et al., 2014; Nakabayashi et al., 2005; Zhou et al., 2018; Corbett et al., 2015; Kuschal et al., 2016; Theil et al., 2017; Kuo et al., 2019; Theil et al., 2019; Botta et al., 2021; Iben et al., 2002). A comprehensive examination of the literature involving 112 individuals diagnosed with trichothiodystrophy revealed neurological abnormalities in a significant proportion of cases (86%) (Faghri et al., 2008). These individuals exhibited various neurological complications, including intellectual impairment (75%), developmental delay (68%), microcephaly (50%), impaired motor control or psychomotor retardation (37%), ataxia (26%), demyelination of cortical neurons (14%), and cerebral atrophy (4%).

### 4.4 Fanconi Anaemia (FA) pathway related syndromes/diseases

Fanconi Anaemia is a rare genetic disease characterized by impaired cell cycle regulation and DNA repair mechanisms (Moreno et al., 2021). The FA-BRCA pathway (FA pathway), involving various *FANC* genes, is essential for DNA damage response, particularly in repairing DNA inter-strand crosslinks, resulting in genome instability characterized by chromosomal breaks and radial figures (Su and Huang, 2011). In brain development, the FA pathway ensures genomic integrity and NPCs survival during replicative stress (Sii-Felice et al., 2008; Forrer Charlier and Martins, 2020). While BRCA1 contributes significantly to inhibition of apoptosis in NPCs (Pao et al., 2014), FANCA is necessary for the maintenance and survival of neural stem cells (NSCs), contributing to the proper brain development (Sii-Felice et al., 2008).

Genetically, FA is characterized by the defects in one or multiple of the 23 FANC proteins (FA subtype proteins) from FANCA through FANCY The disease follows autosomal recessive pattern with 21 genes involved, including: *FANCA*, *FANCC*, *FANCD1/BRCA2*, *FANCD2*, *FANCE*, *FANCF*, *FANCG/XRCC9*, *FANCI*, *FANCJ/BRIP1*, *FANCL*, *FANCM*, *FANCN/PALB2*, *FANCO/RAD51C*, *FANCP/SLX4*, *FANCQ/ERCC4*, *FANCS/BRCA1*, *FANCT/UBE2T*, *FANCU/XRCC2*, *FANCV/REV7* and *FANCW/RFWD3*, *FANCY/FAP100* (Ceccaldi et al., 2016; Niraj et al., 2019; Lipton et al., 2020; Moreno et al., 2021; Repczynska et al., 2022). Additionally, FA can also exhibit an X-linked recessive pattern caused by *FANCB* (Meetei et al., 2004), and *de novo* autosomal dominant pattern by *FANCR/RAD51* (Ameziane et al., 2015; Wang et al., 2015; Repczynska et al., 2022).

In human, mutations in *FANCA*, *FANCC*, and *FANCG* genes account for 80 to 90 percent of FA cases, with *FANCA* mutation being detected in 70% of cases (Moreno et al., 2021). These mutations have been associated with an increased incidence of CNS abnormalities, including ectopic neurohypophysis, adenohypophysis hypoplasia, platybasia, and other abnormalities in the midline of the skull base and posterior fossa (Pavlakis et al., 1992; Deepak Amalnath et al., 2012).

A study by Shahin et al. (2023) revealed a direct association between the FA pathway and the Ncf1/Igfbp2 signalling in regulating NSC fate. They found that inhibiting *Brca1* DNA repair genes in murine *Ncf1* ^*−/−*^ and *Igfbp2* ^*−/−*^ *NPCs* resulted in the exit from self-renewal to neurogenesis, akin to WT NSCs. Conversely, *Fanca* overexpression in WT NSCs reduced neurosphere formation, emphasizing the FA pathway’s role in NSC lineage commitment and neurosphere formation. In another study, *Fanca* ^*−/−*^ and *Fancg* ^*−/−*^ mouse embryos demonstrated microcephaly as a result of elevated apoptosis in proliferating NPCs, driven by increased chromosomal instability. Both embryonic and adult Fanconi murine NSCs showed reduced *in vitro* self-renewal capacity and decreased neuron production, underscoring the FA pathway’s importance in regulating neural progenitor self-renewal, proliferation during embryogenesis, and adult brain homeostasis (Sii-Felice et al., 2008).

### 4.5 Microcephaly-associated mutations leading to mitotic spindle defects

Disruptions in brain size can also be caused by mutations in genes participating in mitosis regulation (Bartkowska et al., 2018; Javed et al., 2018). Among these are *KNL1*, *ASPM*, and *CITK,* which are especially required during neurogenesis being extensively expressed in the VZ and SVZ, are known to induce the accumulation of DNA lesions and microcephaly when defective (Silver et al., 2010; Mao et al., 2016; Shi et al., 2019).

Mutations in the kinetochore scaffold 1 (*KNL1*) gene, also known as *MCPH4,* have been described (Fitze et al., 1999; Genin et al., 2012; Saadi et al., 2016; Szczepansk et al., 2016; Zarate et al., 2016). This was endorsed by further studies demonstrating highest expression of KNL1 in the fetal brain at the 9th gestational week, which marks the onset of neurogenesis, and sharply decreasing after birth (Shi et al., 2017). In hESC-derived NPCs, a *KNL1* mutation (*KNL1* ^*c.6125G > A*^) has been associated to aneuploidy, reduced proliferation, premature differentiation, apoptosis, and an abrogated spindle assembly checkpoint (SAC), culminating in microcephaly (Javed et al., 2018). It is known that KNL1 plays an important role during mitosis participating in proper kinetochore assembly, chromosome alignment and SAC signaling (Ahn and Jun, 2007). Thus, when KNL1 is defective, the SAC can be prematurely deactivated, leading to segregation errors and consequently numerical aneuploidy (Silió et al., 2015). Previous studies have reported that altered SAC can cause microcephaly (Genin et al., 2012; Mirzaa et al., 2014). However, recent findings have demonstrated that *Knl1*-induced microcephaly in mice (*Knl1* ^*fl/fl*^; *hGFAP-Cre*) is not a direct result of aneuploidy, but from the generation of missegregated chromosomes carrying DNA damage in the form of DSBs after SAC disruption. These lesions triggered p53-dependent apoptosis, leading to a robust removal of damaged NPCs through microglial phagocytosis (Shi et al., 2019). However, defects in centrosomal proteins can impair brain development without leading to DSBs accumulation, highlighting the differences between SAC disruption and centrosomal defects in microcephaly (Insolera et al., 2014; Shi et al., 2019).

Abnormal spindle-like microcephaly-associated (*ASPM*) localizes to spindle poles during mitosis. It is arguably the most well-known centrosomal gene causative of microcephaly, as recessive mutations cause the MCPH5 syndrome, which is the most prevalent form of MCPH, characterized by severe microcephaly (Létard et al., 2018). Mechanistically, ASPM regulates mitotic spindle formation and localization during neurogenesis, and *ASPM* mutations lead to several mitotic defects in NECs, including abnormal spindle orientation leading to a reduction in the pool of these progenitors (Fish et al., 2006). Furthermore, ASPM was found to promote HR-mediated repair of DSBs following replication stress and UV irradiation (Xu et al., 2021; Wu et al., 2022). Additionally, while ASPM dysfunction induces DNA damage and postnatal p53-dependent apoptosis of cerebellar progenitors in mice, KO of *ASPM* in the ferret has been shown to induce premature displacement of ventricular RGCs to the outer SVZ, suggesting that ASPM regulates cortical expansion (Williams et al., 2015; Johnson et al., 2018; Zhou et al., 2020).

Another MCPH-related protein that localizes to the spindle poles during mitosis is the multi-domain citron kinase (CITK). It has recently also been linked to a syndromic type of microcephaly in humans, known as *MCPH17*, which is marked by reduced brain size, intellectual disability ranging from moderate to severe, short stature, and renal agenesis (Harding et al., 2016). CITK, together with ASPM controls spindle orientation and cytokinesis and its absence results in perturbed spindle orientation and neurogenic cytokinesis of neuroepithelial progenitors (Basit et al., 2016). However, CITK is also responsible for recruiting BRCA1 to DNA damage sites (Iegiani et al., 2022), and interacts with proteins implicated in the HR pathway such as KIF4A and CDKN1B, indicating that it could also participate in the DDR (Harding et al., 2016; Bianchi et al., 2017). In mice, *Citk* deficiency is associated with cytokinesis defects, DSBs accumulation, and p53-dependent apoptosis, and overall impaired mouse neurogenesis leading to microcephaly (Di Cunto et al., 2000; Sarkisian et al., 2002; Bianchi et al., 2017; Iegiani et al., 2022).

### 4.6 Mutations in the EJC complex leading to microcephaly

Neurodevelopmental deficits have also been found in patients presenting mutations in the exon junction complex (EJC) (Laumonnier et al., 2010; Albers et al., 2012; Nguyen et al., 2013), a regulator of mRNA metabolism of which the core complex is constituted by EIF4A3, MAGOH, RBM8A and CASC3 proteins (Gerbracht et al., 2020). The first evidences associating the EJC and brain development came from studies implicating *Magoh* in the occurrence of microcephaly in mice (Silver et al., 2010). Centrosomal defects and extensive DNA damage leading to cell death could be observed as a result of prolonged mitosis, demonstrating that *Magoh* is a crucial regulator of NSC division being required for proper orientation of the mitotic plane and cell fate determination (Silver et al., 2010; Mao et al., 2016). Indeed, the generation of a conditional KO revealed that NSC-specific depletion of *Magoh* induces microcephaly, suggesting that NSC dysfunction and altered mitosis may influence neuronal and stem cell production (McMahon et al., 2014). In general, *Magoh* deficient mice show decreased thickness of all cortical layers, NSC depletion, reduced number of intermediate progenitors, and apoptosis of newborn neurons, all of which lead to microcephaly (Silver et al., 2010; McMahon et al., 2014; Pilaz et al., 2016).

The *RBM8A* gene, also known as *Y14*, is another essential neurogenesis regulator, is located on 1q21.1 and microdeletions within this region are associated to a wide range of human diseases, including microcephaly (Brunetti-Pierri et al., 2008; Mao et al., 2015). In mice, depletion of *Rbm8a* revealed a microcephaly phenotype similar to the one observed in *Magoh*. Here, the neuronal loss is mainly observed in upper cortical layers, and reduced proliferation accompanied of premature differentiation of NPCs were found (Mao et al., 2015; Zou et al., 2015; Mao et al., 2016). *Rbm8a* deficiency can also impair ciliogenesis, provoke centrosome aberrations and cause DNA damage, inducing p53 activation (Silver et al., 2010; Chuang et al., 2019; Kwon et al., 2021). Besides mRNA metabolism, a role for RBM8A in the DDR has recently been described, as it was shown to interact with Ku70/80 (Chuang et al., 2019; Tsai et al., 2021).

Similarly to *Rbm8a* and *Magoh*, *Eif4a3-*deficient mice presented microcephaly accompanied with NSC mitotic defects, DNA damage, and extensive neuronal p53-dependent apoptosis. The p53 activation is described as a major mechanism implicated in EJC-induced microcephaly, as it rescues the brain size phenotype in all *Eif4a3*, *Magoh* and *Rbm8a* mutants when it is inactive (Mao et al., 2016; Bowen and Attardi, 2019; Chuang et al., 2019). As summarized here, EJC components can impair brain development by different mechanisms, including spontaneous DNA damage generation, which is partially responsible for the occurrence of microcephaly in these models.

## 5 Environmental exposures leading to DNA damage-associated microcephaly

Besides genetic mutations, exposure to environmental factors during (early) pregnancy can lead to primary microcephaly. Here we discuss two of the most critical factors associated with fetal DNA damage: ionizing radiation and Zika virus.

### 5.1 Ionizing radiation

The primary mechanism by which IR can injure cells is mainly through DNA damage (Santivasi and Xia, 2014). IR-induced DNA damage can be either direct by causing DNA breaks, especially DSBs, or indirect by producing free radicals, leading to several injuries such as AP sites, SSBs and DSBs. These lesions can induce cell death if not correctly repaired (Borrego-Soto et al., 2015). Despite the indisputable importance of IR use for clinical purposes, many health consequences have been attributed to IR, primarily when the exposure occurs during the embryonic development (Inouye, 1995; Verreet et al., 2016a). Epidemiological studies based on the outcomes of the nuclear bombings of Hiroshima and Nagasaki have shown that exposure to moderate and high doses of IR, especially between weeks 8 and 15 of pregnancy, can have severe consequences, especially for the developing brain inducing long-term neuronal effects (Otake and Schull, 1993; Otake and Schull, 1998). Early exposure to IR has been associated with microcephaly, dementia, affected memory and learning skills, and an increased risk for seizures (Verreet et al., 2016b; Mouton et al., 2021).

Rodent models have been mostly used to supplement human data to elucidate how IR affects brain development. Different studies of *in-utero* irradiated mice showed a reduction in the brain size with a dose threshold of around 0.3 Gy at the onset of neurogenesis (Verreet et al., 2016a; Shimada et al., 2016; Mfossa et al., 2020). When analyzing mouse embryos irradiated at embryonic day (E) 12 onwards, the VZ and SVZ are the central affected brain regions with NPCs undergoing apoptosis, a mechanism underlying microcephaly in these models (Lee et al., 2001; Gatz et al., 2011; Roque et al., 2012; Saha et al., 2014). Accordingly, a reduced expression of the *ASPM* gene in the VZ of E12 irradiated mice was described, inducing the switch from proliferative to neurogenic divisions causing microcephaly through a reduction in the number of NPCs in the VZ (Fujimori et al., 2008). Mitotic defects were also reported as another mechanism underlying IR-induced microcephaly, as mouse embryos irradiated at E13.5 presented centrosomal aberrations culminating in NPC depletion (Shimada et al., 2016). Besides apoptosis and mitotic defects, p53-dependent premature differentiation of NPCs was proposed as another IR-dependent mechanism leading to microcephaly, this time in mice irradiated at E11, an early time point of neurogenesis (Mfossa et al., 2020). Of interest, another study in mouse embryos irradiated at E11 showed that the cortical plate constituted of differentiating neurons was the most affected region (Verreet et al., 2016a), contradicting the hypothesis that proliferative cells are the most radiosensitive ones (Bergonié and Tribondeau, 1959; Verreet et al., 2015).

Despite the indisputable relevance of the previously mentioned mechanisms, the DNA damage resulting in p53-dependent apoptosis is believed to be the main factor leading to microcephaly after radiation exposure (Verheyde et al., 2006; Quintens et al., 2015; Verreet et al., 2015; Mfossa et al., 2020), and as well in many other developmental syndromes (Bowen and Attardi, 2019) as discussed here. Indeed, it is known that IR induces a dose-dependent DSB foci formation in the cerebral cortex of mice, accompanied by a cell cycle arrest at the G2/M checkpoint and p53-dependent apoptosis (Gatz et al., 2011; Saha et al., 2014; Mfossa et al., 2020). Interestingly, p53 is highly expressed in the embryonic brain compared to the adult brain. While it regulates NPC differentiation (Mendrysa et al., 2011), it seems to be dispensable for normal brain development (Wang et al., 2009), even though a small fraction of *Trp53* null mice display exencephaly (Armstrong et al., 1995; Sah et al., 1995). Nevertheless, in the case of a p53 hyperactivation in response to DSB formation following irradiation during embryogenesis, p53 can induce massive apoptosis of NPCs and reduced proliferative states in the developing brain culminating in a range of developmental defects including microcephaly (Bowen and Attardi, 2019; Mfossa et al., 2020).

### 5.2 Viral-induced acquired microcephaly

To date, several viral infections have been linked to microcephaly during pregnancy. For instance, the cytomegalovirus, herpes simplex virus, rubella virus, and Zika virus (ZIKV) can infect the fetus by crossing the placenta, and also present a tropism for the brain, being commonly associated with reduced brain size (Devakumar et al., 2018). Cytomegalovirus is known to alter the progenitor pool fate, induce premature differentiation, and also impair neuronal differentiation through PPARɣ activation (Devakumar et al., 2018; Sun et al., 2020). Herpes simplex virus infection has been linked to NPC pool depletion and impaired differentiation (Krenn et al., 2021). The mechanisms by which Rubella virus lead to microcephaly is still unclear (Devakumar et al., 2018). Here we focus on the most notable virus linked to microcephaly in newborns, the ZIKV.

The ZIKV was first described in Africa in 1947, and subsequent cases were reported in other countries. ZIKV is transmitted through the bite of the infected *Aedes aegypti* mosquito, and in most cases, the infection is not alarming. However, accumulating evidence has shown that infection during pregnancy can lead to fetal genetic neuroabnormalities (Antoniou et al., 2020). It is known that a single aminoacid substitution in prM, one of the ten ZIKV-encoded proteins, is responsible for a change in the virus tropism, specifically targeting the brain (Saade et al., 2020). Despite the fact that ZIKV neurotropism has been known since its discovery, it was not associated to developmental disorders until a few years ago (Ledur et al., 2020). In 2015, ZIKV was declared a Public Health Emergency in Brazil, strongly linked to devastating effects on fetal neurodevelopment, leading to a reduction in brain size (Kindhauser et al., 2016). In response to this outbreak, widespread efforts have been made to understand how ZIKV infection may induce microcephaly. It is known that the interaction between viruses and their hosts can often induce the evolutionarily response to DNA damage, that can be activated upon viral interaction with the host DNA or by ROS production during viral replication (Luftig, 2014; Ryan et al., 2016). Here, we highlight the ZIKV-induced DNA damage and its relation with microcephaly.

It is a consensus in the literature that ZIKV can directly infect human NPCs *in vitro*, mimicking microcephaly (Wen et al., 2017). Different studies revealed an impaired cell cycle progression causing a reduction in human NPC proliferation marked by a reduced expression of the proliferation markers Edu and Ki67, a reduced cell density and cell cycle arrest in S-phase (Cugola et al., 2016; Souza et al., 2016; Tang et al., 2017). These findings were confirmed by different studies using mouse models, that found a reduction in the thickness of the brain cortex and in the number of mitotic cells in the VZ and SVZ, centrosomes amplification and cell cycle arrest in S-, G1- or G2- phases (Li et al., 2016; Nguyen et al., 2016; Shao et al., 2016; Wu et al., 2016). Besides cell cycle impairments, premature neuronal differentiation (Gabriel and Gopalakrishnan, 2017; Saade et al., 2020) and cell death (Wen et al., 2017) are also frequently found to contribute to ZIKV-induced microcephaly, although so far only the latter has been clearly shown to be associated with DNA damage induction. Post-mortem analysis of fetal brain tissues revealed cortical thinning and extensive apoptosis (Driggers et al., 2016), and different studies using human iPSC-derived NPC, brain organoids and mouse models reaffirmed these findings, showing increased cell death marked by caspase 3 activation in the neocortex and DNA fragmentation leading to a reduction in the NPC pool (Cugola et al., 2016; Garcez et al., 2016; Qian et al., 2016; Miner et al., 2016; Shao et al., 2016; Souza et al., 2016; Wu et al., 2016; Tang et al., 2017). The effects of ZIKV on astrocytes, a cell type with the highest infection rate in the brain, were also demonstrated showing mitochondrial damage and ROS production leading to DNA damage followed by cell death (Ledur et al., 2020).

It is known that virus-induced DNA damage can generate lesions such as SSBs and DSBs (Ryan et al., 2016). RNA-seq analysis showed *BRCA1* and *MRE11A* upregulation in neurospheres infected with ZIKV, and an increased γH2A.X signal has also been demonstrated in NPCs, both indicating a possible DDR to DSBs after ZIKV infection (El Ghouzzi et al., 2016; Garcez et al., 2017). Further studies have validated these findings showing that ZIKV can induce the DDR upon DSBs recognition contributing to NPC depletion (Hammack et al., 2019). Hammack et al. (2019) reported ATM/Chk2 pathway activation in response to DSBs in infected NPCs, and also showed inhibited cell cycle progression through S-phase which seems to provide a beneficial environment to Uganda and Puerto Rico ZIKV strains replication and thereby restricted NPC proliferation. Of interest, while both strains induced cell death in NPCs, in NSCs a distinct infection pattern was found for both strains. In particular, the Uganda strain increased yH2A.X levels, whereas the Puerto Rico strain arrested cell cycle progression through elevated p53 levels. It was observed that NSCs infected by the Puerto Rico strain when stimulated to differentiate into progenitors presented higher susceptibility to cell death (Devhare et al., 2017). In the Brazilian ZIKV strain, Cyclin E downregulation and Cyclin-dependent kinase inhibitor 1A upregulation have been pointed as another mechanism by which ZIKV induces cell cycle arrest and reduces the NPC pool (Garcez et al., 2017). Interestingly, NSC-derived NPCs infected with the Uganda strain presented less viral replication when compared to parental NSCs, which can partly explain the severity of ZIKV infection during early brain development (Devhare et al., 2017). Recently, a link between PNKP inhibition and ZIKV infection has been identified. Here, PNKP depletion led to DNA damage accumulation in NPCs after ZIKV infection, failure to activate the DNA damage checkpoints Chk1 and Chk2 and cytoplasmic accumulation of CycA/CDK1 complexes resulting in mitotic catastrophe (Rychlowska et al., 2022).

Global genetic analyses trough RNA-seq revealed a downregulation of cell cycle-related and DDR pathways, including many p53-dependent genes, upon ZIKV infection. NER, MMR, HR, ATR and G2/M checkpoint were found downregulated after infecting human neural crest cells and neurons with the Asian strain, while a couple of downregulated genes implicated in HR, NHEJ and FA pathways were found after infection of NPCs with the Asian and African strains (El Ghouzzi et al., 2016; Zhang et al., 2016; Park et al., 2020). These analyses also found an enrichment of downregulated genes that encode centrosomal proteins which are causative of microcephaly (Marthiens et al., 2013).

## 6 The central role of p53 activation in the etiology of DNA damage-associated microcephaly

**FIGURE 2:**
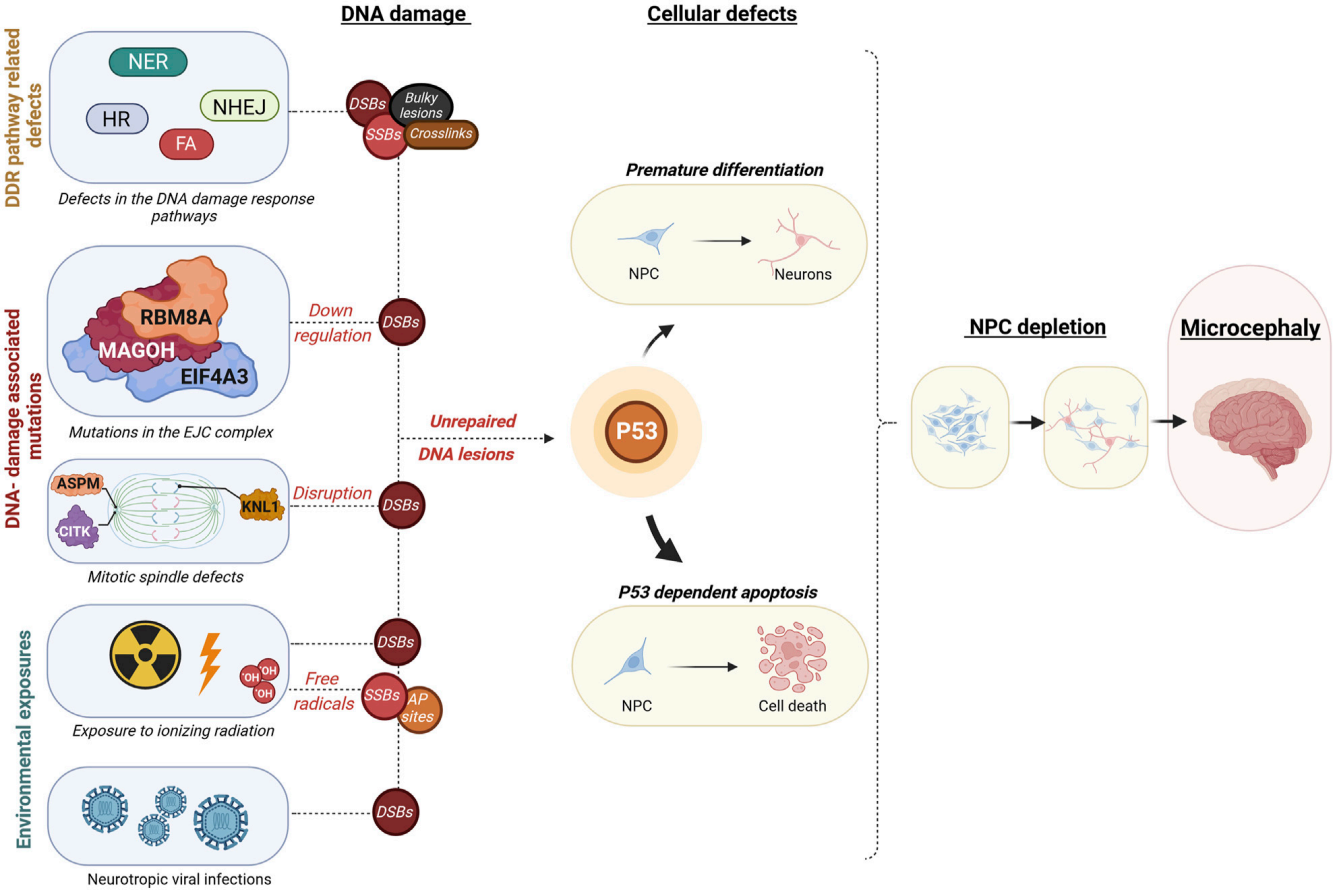
Overview of mechanisms leading from embryonic DNA damage accumulation to microcephaly. This diagram outlines events that may lead to an accumulation of DNA damage during embryonic development, leading to microcephaly. These disruptions could be due to inherent genetic mutations within constituents of DDR pathways such as in Nucleotide excision Repair (NER), Non-homologous end joining (NHEJ), Homologous recombination (HR), or Fanconi anaemia (FA) pathways, resulting in different types of DNA damage as double-strand breaks (DSB), single-strand breaks (SSB), Aapurinic/apyrimidinic (AP) sites, bulky lesions, or crosslinks. Additionally, DSB accumulation may result from disruptions in the core exon-junction complex (EJC) proteins RNA binding motif protein 8A (RBM8A), Mago homolog (MAGOH), Eukaryotic translation initiation factor 4A3 (EIF4A3) or proteins involved in mitotic spindle assembly such as Abnormal spindle microtubule assembly (ASPM), Citron rho-interacting kinase (CITK), Kinetochore scaffold 1 (KNL1). Environmental factors leading to excessive DNA damage are fetal exposure to high doses of ionizing radiation or fetal neurotropic viral infections such as the Zika virus. These defects may ultimately cause a buildup of uncorrected DNA lesions, which instigates a cascade of events encompassing mainly cell death, but also in some cases premature differentiation of neural progenitor cells. The protein p53 plays a crucial role in coordinating these responses at the cellular level, which can ultimately lead to a decrease in brain size, a condition known as microcephaly. Illustration created using Biorender (https://www.biorender.com/).

From all of the above, it can be concluded that one of the main converging mechanisms underlying the most severe forms of microcephaly is the inappropriate hyperactivation of p53 (Figure 2) during early stages of neurodevelopment (Bowen and Attardi, 2019; Tsai et al., 2021). Indeed, in many of the animal models described above, the reduction in brain size can at least be partially rescued by knocking out *Trp53*. In that respect, it should not be too surprising that inhibiting mutations in *ATM*, the primary regulator of p53 in response to DSBs, and *TP53* itself do not lead to microcephaly. Moreover, based on transcriptomic studies performed in some of these experimental models, including genetic models (e.g., *Magoh*, *Citk*, *Knl1*, *Ino80*) as well as those exposed to ionizing radiation and ZIKV, it is clear that p53 is a major regulator of the transcriptional response and core signatures of genes seem to be shared between them (El Ghouzzi et al., 2016; Quintens, 2017; Mitchell-et al., 2019). Nevertheless, they also have their specific gene signatures and phenotypic outcomes.

What exactly determines which genes are activated by p53 under which specific circumstances remains to be determined. Three factors might be of particular importance: (Tubbs and Nussenzweig, 2017) the dynamics (i.e., amplitude and duration) of p53 activation, (Mikolaskova et al., 2018) the exact developmental timing of p53 activation, and (Senturk and Manfredi, 2013) the cell/tissue type in which p53 becomes hyperactivated, which may be linked to either cell/tissue-type specific expression of p53 or p53 post-translational modifications (Hamard et al., 2013; Resnick-Silverman et al., 2023) or isoforms (Joruiz and Bourdon, 2016). Bowen et al. (2019) used conditional alleles to artificially activate p53 to various degrees in specific embryonic cell types at different developmental stages to recapitulate some p53-driven developmental syndromes. This study showed that p53 hyperactivation primarily affected neuronal crest cell derivatives, providing a further basis for neurodevelopmental phenotypes like microcephaly. One of the most apparent reasons for the particular sensitivity of the embryonic brain to the effects of DNA damage, e.g., after irradiation, might be the inherently high potential for high p53 activity in the embryonic mouse brain compared to other (embryonic) tissues (Komarova et al., 1997). Also, NECs have a lower threshold for apoptosis compared to cells generated in later developmental stages (Lee et al., 2012a; Lee et al., 2012b; McKinnon, 2013), and the shorter G1 phase in NECs compared to NPCs renders them more susceptible to genotoxic stress (Kalo et al., 2019). Furthermore, radiation-induced DNA damage results in a stronger p53-mediated response in NPCs and immature neurons, compared to more differentiated cells (Martin et al., 2009; Quintens et al., 2015; Mfossa et al., 2020). This may explain why the brain, specifically the proliferating cells populating it, is the most sensitive organ to DNA damage during embryonic development.

## 7 Conclusion and perspectives

Microcephaly is a rare neurodevelopmental disorder with heterogeneous causes. Compensating the difficulty of studying this condition in humans, animal models resembling genetic and non-genetic models opened doors to further investigate the mechanisms behind microcephaly. These studies reinforced the understanding of the high sensitivity of the developing brain to DNA damage, highlighting the importance of a functional DDR for proper cortical development, and revealed different mechanisms accounting for the reduced brain size, mainly culminating in p53-dependent apoptosis and NPC pool depletion due to DSB accumulation. Despite the enormous efforts made to understand microcephaly etiology, its underlying mechanisms should be further investigated. Studies on the DNA damage effects in other NPC types such as the oRGCs, abundant in the embryonic human brain and scarce in the mouse, could be interesting to obtain more insights on the specific mechanisms underlying microcephaly in humans. Recent developments using human brain organoid cultures, which can be derived from patient stem cells or exposed to radiation or ZIKV, may prove extremely useful as experimental models for further research (Gabriel et al., 2020). For instance, a recent study applied a CRISPR-Cas9 screening method of 172 microcephaly candidate genes combined with cellular lineage tracing in human organoids. It confirmed the DNA damage response as one of Field’s most critical pathways (Esk et al., 2020).

Another outstanding question related to the role of p53 that needs further investigation is what determines the cell’s fate, whether it be apoptosis or premature differentiation. Can this be linked to cell-specific activation of specific gene signatures, does it depend on the cell cycle stage during which p53 activation occurs? Furthermore, other p53-dependent and–independent mechanisms besides apoptosis and differentiation may underpin neurodevelopmental defects, including defective neuronal migration, senescence or neuroinflammation. Finally, most research on microcephaly so far has focused on glutamatergic neurogenesis, although also interneuron progenitors are sensitive to p53 hyperactivation after EJC dysfunction (Sh et al., 2020) or irradiation (Mfossa et al., 2020). It will be necessary to uncover the contribution of interneuron depletion to microcephaly syndromes, mainly since they can be associated with epilepsy or seizures.
